# TFE3 activation in a *TSC1*‐altered malignant PEComa: challenging the dichotomy of the underlying pathogenic mechanisms

**DOI:** 10.1002/cjp2.187

**Published:** 2020-11-12

**Authors:** Maren Schmiester, Anna Dolnik, Uwe Kornak, Berit Pfitzner, Michael Hummel, Denise Treue, Arndt Hartmann, Abbas Agaimy, Veronika Weyerer, Anja Lekaj, Susanne Brakemeier, Robert Peters, Robert Öllinger, Sven Märdian, Lars Bullinger, Jana Käthe Striefler, Anne Flörcken

**Affiliations:** ^1^ Department of Hematology, Oncology, and Tumor Immunology Charité‐Universitätsmedizin Berlin, Corporate Member of Freie Universität Berlin, Humboldt‐Universität zu Berlin, and Berlin Institute of Health Berlin Germany; ^2^ Berlin Institute of Health (BIH) Berlin Germany; ^3^ Institute of Medical Genetics and Human Genetics Charité‐Universitätsmedizin Berlin, Corporate Member of Freie Universität Berlin, Humboldt‐Universität zu Berlin, and Berlin Institute of Health Berlin Germany; ^4^ Institute of Human Genetics University Medical Center Göttingen Göttingen Germany; ^5^ Institute of Pathology DRK Kliniken Berlin Westend Berlin Germany; ^6^ Institute of Pathology Charité‐Universitätsmedizin Berlin, Corporate Member of Freie Universität Berlin, Humboldt‐Universität zu Berlin, and Berlin Institute of Health Berlin Germany; ^7^ Institute of Pathology Friedrich‐Alexander‐University Erlangen‐Nürnberg, University Hospital Erlangen Erlangen Germany; ^8^ Department of Nephrology and Medical Intensive Care Charité‐Universitätsmedizin Berlin, Corporate Member of Freie Universität Berlin, Humboldt‐Universität zu Berlin, and Berlin Institute of Health Berlin Germany; ^9^ Department of Urology Charité‐Universitätsmedizin Berlin, Corporate Member of Freie Universität Berlin, Humboldt‐Universität zu Berlin, and Berlin Institute of Health Berlin Germany; ^10^ Department of Surgery Charité‐Universitätsmedizin Berlin, Corporate Member of Freie Universität Berlin, Humboldt‐Universität zu Berlin, and Berlin Institute of Health Berlin Germany; ^11^ Center for Musculoskeletal Surgery Charité‐Universitätsmedizin Berlin, Corporate Member of Freie Universität Berlin, Humboldt‐Universität zu Berlin, and Berlin Institute of Health Berlin Germany; ^12^ German Cancer Consortium (DKTK), Partner Site Berlin, and German Cancer Research Center (DKFZ) Heidelberg Germany

**Keywords:** PEComa, TFE3, TSC1, whole genome sequencing, RNA sequencing, FISH

## Abstract

Perivascular epithelioid cell tumors (PEComas) form a family of rare mesenchymal neoplasms that typically display myomelanocytic differentiation. Upregulation of mTOR signaling due the inactivation of *TSC1/2 (Tuberous Sclerosis 1 and 2)* is believed to be a key oncogenic driver in this disease*.* Recently, a subgroup of PEComas harboring *TFE3* (*Transcription Factor E3*) rearrangements and presenting with a distinctive morphology has been identified. *TSC1/2* and *TFE3* aberrations are deemed to be mutually exclusive in PEComa, with two different pathogenic mechanisms assumed to lead to tumorigenesis. Here, we challenge this dichotomy by presenting a case of a clinically aggressive *TCS1*‐mutated PEComa displaying a *TFE3*‐altered phenotype. FISH analysis was suggestive of a *TFE3* inversion; however, RNA and whole genome sequencing was ultimately unable to identify a fusion involving the gene. However, a copy number increase of the chromosomal region encompassing *TFE3* was detected and transcriptome analysis confirmed upregulation of *TFE3,* which was also seen at the protein level. Therefore, we believe that the TSC1/2‐mTOR pathway and TFE3 overexpression can simultaneously contribute to tumorigenesis in PEComa. Our comprehensive genetic analyses add to the understanding of the complex pathogenic mechanisms underlying PEComa and harbor insights for clinical treatment options.

## Introduction

PEComas are a family of mesenchymal tumors that demonstrate immunoreactivity for both melanocytic and smooth muscle markers [1, 2]. They form a biological continuum ranging from benign to overtly malignant aggressive neoplasms. Malignant PEComas are exceedingly rare, with only about 100 cases described in the literature. Radical resection remains the favored treatment option in localized tumors. In locally advanced or metastatic disease, single case studies report short‐lived responses to chemotherapy containing doxorubicin, ifosfamide or gemcitabine [3].

Germline or somatic mutations of the genes *TSC1* or *TSC2* (*TSC1/2*) are a driving factor in PEComa development, resulting in activation of the mammalian target of rapamycin (mTOR) pathway [4, 5]. These alterations are the basis for palliative therapy with mTOR inhibitors [3].

A subset of PEComas harboring *TFE3* gene fusions has recently been identified, adding them to the group of Xp11 translocation cancers. Here, the introduction of a constitutively active promotor causes oncogenic upregulation of the TFE3 transcription factor [6, 7]. Beside Xp11 translocations, several other causative genomic alterations for TFE3 activation likely exist [8].

Current consensus is that *TFE3* and *TSC1/2* alterations define distinct biological PEComa subgroups and are mutually exclusive [6, 9, 10]. Here, we report for the first time a malignant PEComa with TFE3 activation and heterozygous loss of *TSC1*. This case challenges the molecular dichotomy in this tumor entity.

## Materials and methods

### Studied case

Analyses were performed on tumor samples obtained from a 47‐year‐old woman diagnosed with PEComa in 2017. Written informed consent for the analyses was provided by the patient and posthumously by her husband according to local ethical guidelines.

### Immunohistochemical staining and fluorescence *in situ* hybridization

Samples of the primary tumor and metastases were formalin‐fixed and paraffin‐embedded. H&E stained slides were evaluated by specialized pathologists at Charité‐Universitätsmedizin Berlin and the reference center for urogenital and soft tissue pathology at the Institute of Pathology of the University Erlangen‐Nürnberg in Erlangen, Germany. Immunohistochemistry was performed as described in Supplementary materials and methods using antibodies listed in supplementary material, Table S1.

### Molecular analyses

Quantitative PCR, targeted exome sequencing, RNA sequencing (RNA‐Seq) and nanopore long‐read whole‐genome sequencing was performed as described in Supplementary materials and methods.

## Results and discussion

### Case description

In 2017, a 47‐year‐old woman with known tuberous sclerosis complex (TSC) due to a heterozygous germline *TSC1* deletion presented with abdominal pain. A CT scan revealed a mass in the right kidney, leading to the clinical diagnosis of angiomyolipoma. Everolimus was initiated at a low dose of 2.5 mg once daily in accordance with the patient's wishes, resulting in trough levels of 2.5–4 ng/ml.

Four months later, imaging showed rapid growth of the mass. With suspicion of renal cell carcinoma, a radical nephrectomy was performed. Histopathological examination led to the diagnosis of a PEComa, which was 120 × 110 × 110 mm in size and resected to R0. A CT scan of the lungs showed no pulmonary metastases and the tumor board recommended no adjuvant therapy.

Four months later, recurrence was seen in a follow‐up CT scan in the region T12‐L4. The patient reported no symptoms at this time. Everolimus was re‐initiated at 5 mg twice daily. In addition, sorafenib was administered at 200 mg twice daily but had to be discontinued after 2 months due to intolerable gastrointestinal side effects. The patient also received radiation therapy of the painful paravertebral mass (39–45.5 Gy), leading to pronounced tumor regression.

However, hepatic masses appeared after 12 weeks and histopathology confirmed the diagnosis of metastatic malignant PEComa. The patient underwent surgery with R1 resection and did not return to our clinic for 4 months. By then, multiple hepatic, osseous and pulmonary metastases had developed. Chemotherapy with doxorubicin (75 mg/m^2^) could only be administered once and the patient died of the disease in 2018.

### Distinctive *TFE3*‐rearranged phenotype in a *TSC1*‐altered PEComa

**Figure 1:**
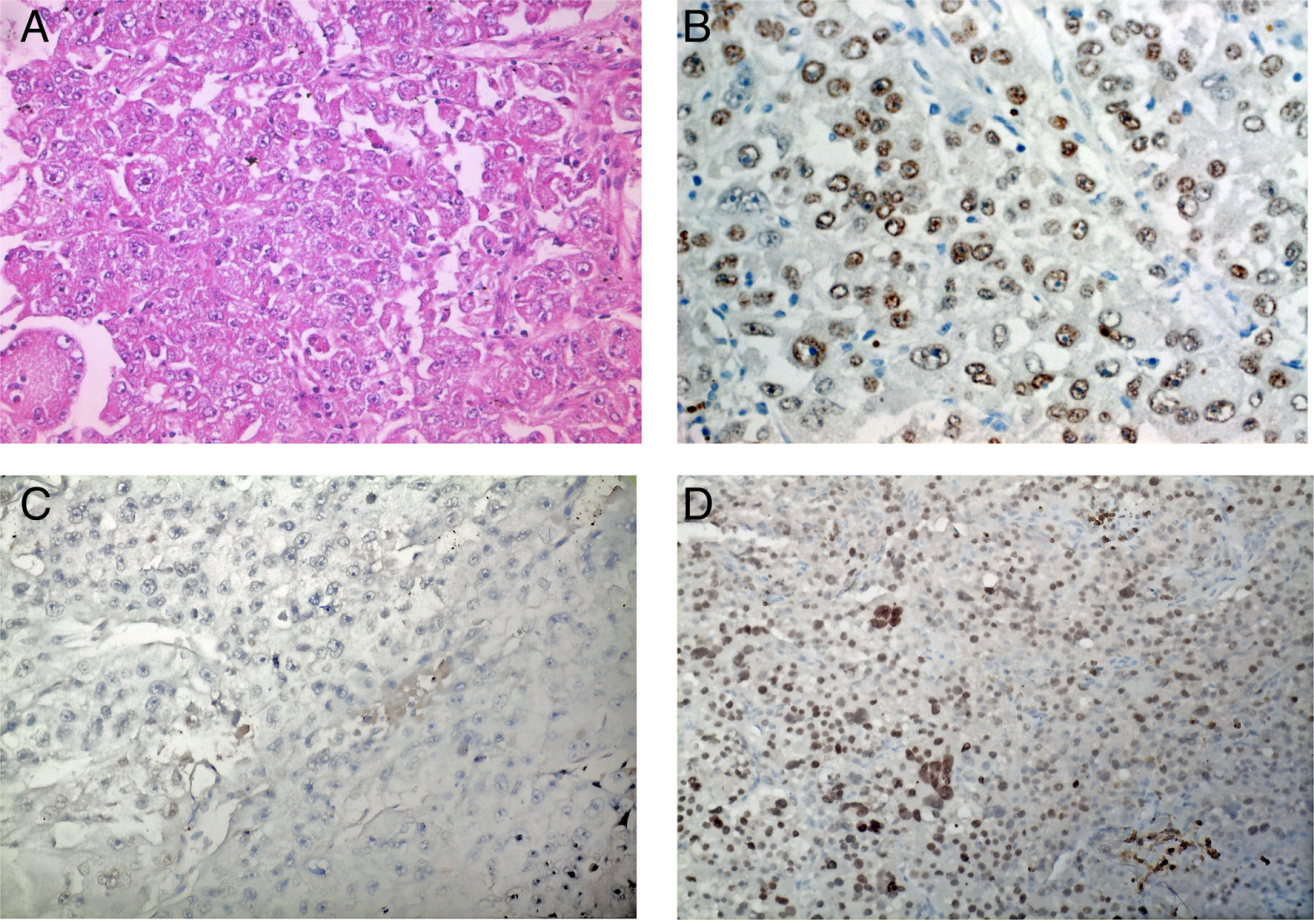
PEComa histology and immunohistochemistry (IHC). (A) Representative image of the histomorphological appearance of the presented case (100‐fold total magnification, H&E staining). (B) TFE3 expression in tumor cells (200‐fold total magnification). (C) No MiTF expression in tumor cells from primary tumor (200‐fold total magnification). (D) MiTF expression in tumor cells from hepatic metastasis (200‐fold total magnification).

Histomorphologically, conventional PEComas (so called epithelioid angiomyolipomas) display a monophasic pattern of predominantly epithelioid cells and lack adipose tissue and dysmorphic blood vessels [11]. TFE3‐rearranged PEComas show epithelioid cells arranged into a distinctive nested and pseudoalveolar pattern [6, 10]. The PEComa presented here matched the latter description, displaying a solid, partially nested‐alveolar architecture with extensive tumor necrosis as well as hemorrhage. Adipose tissue and spindle cells were completely absent. The tumor cells contained voluminous, eosinophilic and partially clear cytoplasm with prominent nucleoli and pleomorphic nuclei. Marked nuclear atypia was present (Figure 1A).

Immunohistochemical examination of the vital tumor cells revealed partial patchy expression of HMB45, focal expression of Melan A and weak positivity for Cathepsin K, which was mirrored at the RNA level (see supplementary material, Figure S1). There was no immunoreactivity for PAX8, smooth muscle actin and desmin and strong nuclear positivity for TFE3 was observed, again matching the phenotype of *TFE3*‐altered PEComas (Figure 1B) [6, 10]. As opposed to the primary tumor, MiTF positivity was seen in hepatic metastases (Figure 1C,D), while pulmonary metastases displayed only weak *MiTF* mRNA expression (see supplementary material, Figure S1). Many *TFE3*‐rearranged PEComas are MiTF‐nonimmunoreactive and it has been suggested that the TFE3 fusion protein substitutes for MiTF [6]. We consider tumor heterogeneity a possible explanation for the differential MiTF expression, with perhaps only a subset of tumor cells exhibiting TFE3 activation.

### Evidence for a dual pathogenic mechanism involving *TFE3* and *TSC1* in our PEComa case

**Figure 2:**
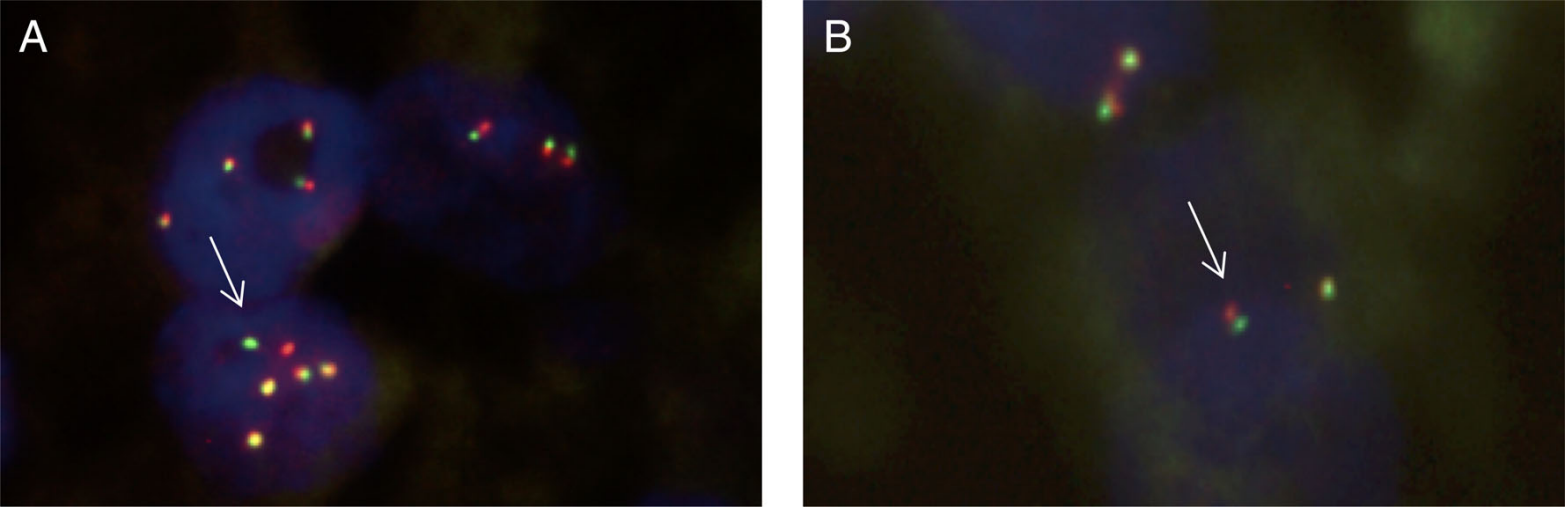
Interphase FISH with *TFE3* break‐apart probe. (A) Break‐apart signal as seen in 6% of cells (arrow). (B) Small separation of the *TFE3* probes (arrow).

**Figure 3:**
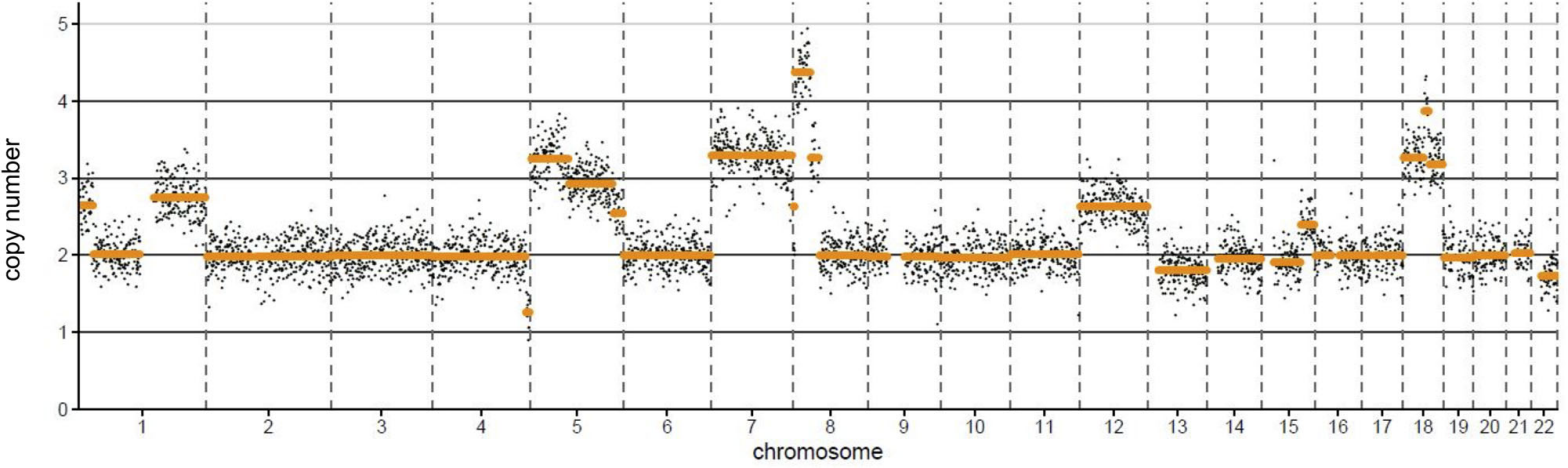
Genomic profiling by an Oxford Nanopore Sequencing‐based assay of PEComa chromosomes 1‐22 finds multiple copy number variations. 2‐Fold genome coverage was achieved; counts averaged over 0.5 MB. Chromosome X is not analyzed or depicted here due to the algorithm used.

**Figure 4:**
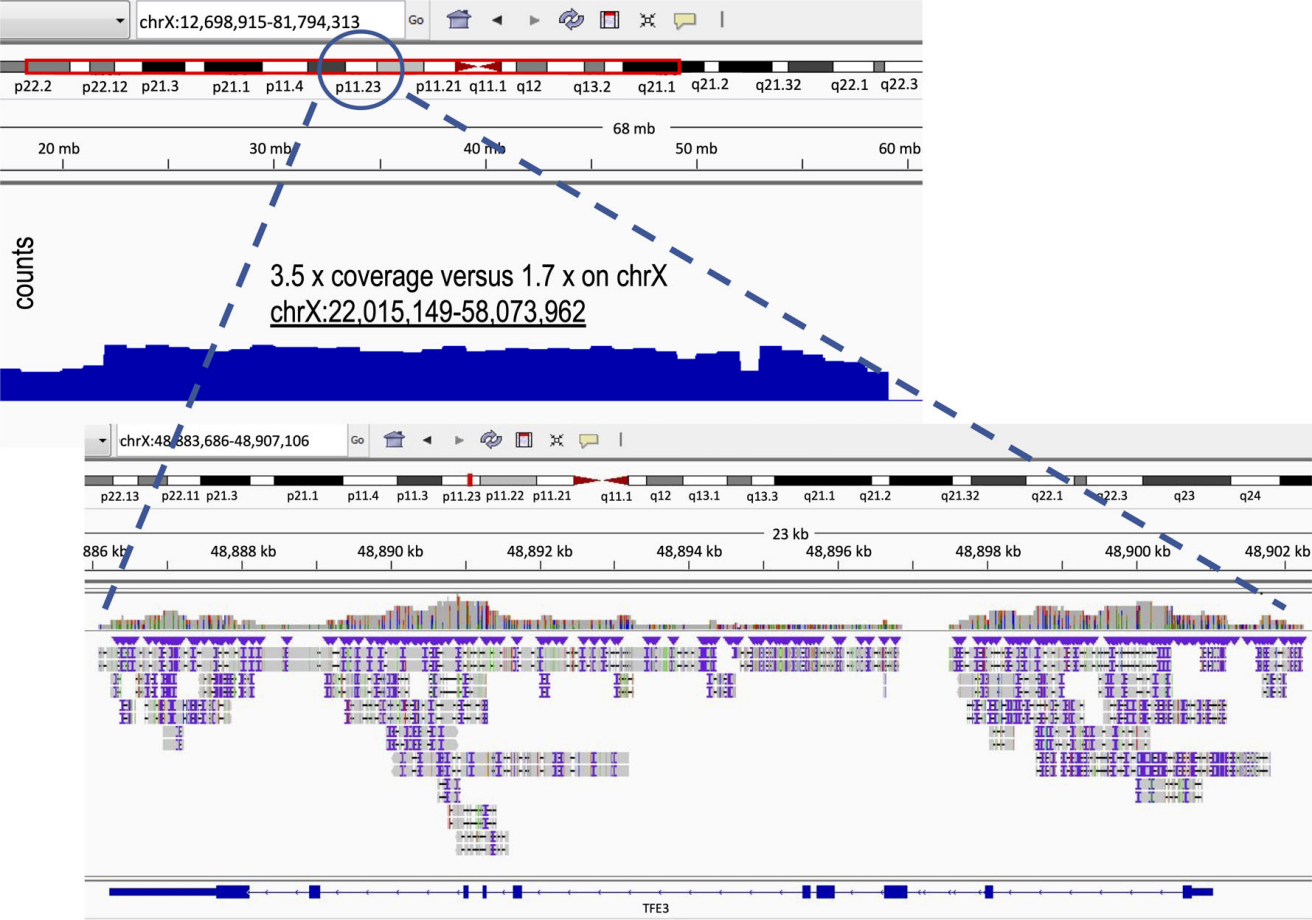
Genomic profiling by an Oxford Nanopore Sequencing‐based assay of PEComa chromosome X finds copy number gain at chrX:22015149‐58073962. 2‐Fold genome coverage was achieved. Copy number variation for chromosome X (top left panel) and enlarged view of copy number changes for *TFE3* (lower right panel).

The known heterozygous germline *TSC1* deletion was verified by real‐time PCR (see supplementary material, Figure S2) and only residual *TSC1* expression was seen in the transcriptome (see supplementary material, Figure S1). The tumor morphology and TFE3 immunoreactivity, however, were suggestive of a *TFE3* fusion. Therefore, fluorescence *in situ* hybridization (FISH) analysis was performed on the primary tumor. Out of 50 tumor cells, only three showed a translocated break‐apart signal (Figure 2A). The other examined cells did not meet the break‐apart cutoff values, but several cells displayed one co‐localization signal and one small separation of the 5′*TFE3* and 3′*TFE3* probes (Figure 2B). Similar subtle break‐apart patterns have been described in tumors harboring *TFE3* inversions rather than translocations [12, 13, 14]. However, RNA‐Seq analysis performed on a pulmonary PEComa lesion was unable to identify any gene fusions involving *TFE3*. In order to find aberrations missed by RNA‐Seq, we next performed long‐read whole genome sequencing. Structural variant calling also did not detect *TFE3* fusions. However, multiple copy number gains were found, including a large 36 Mbp region encompassing *TFE3* on chrX:22015149‐58073962 (Figures 3 and 4). Transcriptome analysis detected an increase in levels of *TFE3* mRNA in tumor cells compared to dermal fibroblast controls after normalization for expression of housekeeping genes (see supplementary material, Figure S1). Therefore, similar to Xp11 translocation cancers, *TFE3* overexpression is one likely oncogenic driver in this tumor [15].

In addition, targeted exome sequencing revealed a coexistent *TP53* mutation. The variant NM_000546.5:c.614A>G (p.Y205C) found was classified as pathogenic. *TP53* mutations do occur concurrently with *TSC1/2* mutations [10] but have not been described in *TFE3*‐altered PEComas until now. No additional pathogenic mutations were detected in the other genes tested in our panel (see supplementary material, Table S2).

## Conclusion and clinical remarks

In the case presented here, it is clear that the heterozygous loss of *TSC1* predisposed to PEComa development, but we believe that the sequential gain of TFE3 contributed to tumorigenesis. Interestingly, the observed TFE3 activation was not due to a *TFE3* gene fusion, analogous to reports on TFE3‐expressing/nontranslocated renal cell carcinomas. These tumors are hypothesized to share a biological mechanism with their translocated counterparts based on a similar morphology, immunolabeling and increased expression of a common RNA read‐through molecule [16, 17]. In some of these neoplasms, *TFE3* amplification leads to increased gene expression, but other mechanisms resulting in elevated TFE3 levels likely also exist [8, 18]. The same presumably holds true for PEComas like the one presented here. Regarding the TFE3 expression in our case, clonal tumor evolution appears to have played an additional role during the course of disease. At initial diagnosis, FISH analysis was unable to detect a *TFE3* amplification in the primary tumor, although genomic profiling of metastases later revealed a copy number gain. Perhaps the additional *TP53* mutation, which presumably also contributed to the complex karyotypic changes, promoted the selection of a TFE3‐expressing PEComa subclone in which genomic material on chromosome X was consecutively gained. Indeed, the differential MiTF expression observed here suggests tumor heterogeneity and supports our hypothesis of clonal tumor evolution.

To our knowledge, this is the first case with confirmed heterozygous deletion in a *TSC* gene and concomitant *TFE3* activation with a distinctive malignant epithelioid phenotype. This finding challenges the biological distinction of *TFE3*‐ and *TCS1/2‐*altered PEComas. The notion that both the TSC1/2‐mTOR pathway and TFE3 overexpression can simultaneously contribute to tumorigenesis in PEComa is of translational clinical importance. *TSC1/2*‐mutated PEComas sometimes respond to mTOR‐inhibition therapy [19], but these drugs are mechanistically believed to be inefficient in *TFE3*‐altered PEComa. MET‐inhibitors, on the other hand, are active in alveolar soft part sarcoma with *TFE3* rearrangement [20], a rare subtype of soft‐tissue sarcoma, and could constitute a therapeutic option for TFE3 overexpressing PEComas. Importantly, when there is evidence of dual pathway activation, it appears reasonable to combine both drugs.

## Author contributions statement

AF, LB, AD, UK and MS designed the research. AD, DT and AL performed the experiments. LB, AD, UK, BMP, VW, AH, AA and MS analyzed and interpreted the data. MS and JKS wrote the manuscript. AF, LB, AD, UK, DT, MH, BP, VW, AH, AA, SB, RP, RÖ and SM critically commented on and edited the manuscript. All authors have read and approved the manuscript.

## Supporting information


**Supplementary materials and methods**
**Figure S1.** Expression levels of candidate genes in tumor tissue detected by RNA‐Seq compared to dermal fibroblasts
**Figure S2.** Results of quantitative PCR for determination of *TSC1* copy number compared to the autosomal *ALB* and the X‐chromosomal *F8* gene loci
**Table S1.** Antibodies used for immunohistochemical staining
**Table S2.** Genes covered in the NGS panel developed for clinical service at Charité – Universitätsmedizin Berlin
