# Supplementary material for: TFE3 activation in a TSC1‐altered malignant PEComa: challenging the dichotomy of the underlying pathogenic mechanisms
Source: J Pathol Clin Res. 2020 Nov 12;7(1):3–9. doi: 10.1002/cjp2.187 (PMC7737753; doi:10.1002/cjp2.187)
Supplement: Supplementary file 1 — Supplementary materials and methods Figure S1. Expression levels of candidate genes in tumor tissue detected by RNA‐Seq compared to dermal fibroblasts Figure S2. Results of quantitative PCR for determination of TSC1 copy number compared to the autosomal ALB and the X‐chromosomal F8 gene loci Table S1. Antibodies used for immunohistochemical staining Table S2. Genes covered in the NGS panel developed for clinical service at Charité – Universitätsmedizin Berlin [file CJP2-7-3-s001.pdf]

TFE3 activation in a *TSC1*-altered malignant PEComa: challenging the dichotomy of the underlying pathogenic mechanisms

Schmiester M *et al.* *J Pathol Clin Res* DOI: 10.1002/cjp2.187

Supplementary Material

Contents

Supplementary materials and methods

Figure S1

Figure S2

Table S1

Table S2

Supplementary materials and methods

Immunohistochemical staining and fluorescence in situ hybridization (FISH)

Whole slides of the tumor block (primary tumor, hepatic and pulmonary metastases) were evaluated. Sections were stained following the manufacturers protocols in laboratories accredited by the German Accreditation Office (DAKKs) according to DIN EN ISO/IEC 17020. Immunohistochemical analysis was performed on a BenchMark *ULTRA* Automated IHC/ISH Slide Staining System (Ventana Medical Systems, Tucson, AZ, USA).

The Zyto*Light* ® SPEC TFE3 Dual Color Break Apart Probe (ZytoVision GmbH, Bremerhaven, Germany) was used to screen for translocations involving the chromosomal region Xp11.23 harboring the *TFE3* gene. FISH was performed following the manufacturer´s instructions.

RNA sequencing

RNA was extracted from fresh frozen pulmonary tumor tissue using the Trizol (Thermo Fisher Scientific, Hennigsdorf, Germany) method, followed by purification using the Direct-zol RNA Mini Prep Kit (Zymo Research, Freiburg, Germany). Polyadenylated mRNA was enriched using the NEBNext Poly(A) mRNA - Magnetic Isolation Module (New England Biolabs, Frankfurt, Germany). The NGS library was prepared using the NEBNext Ultra - RNA Library Prep Kit for Illumina (New England Biolabs, Frankfurt, Germany) and NEB Next Multiplex Oligos for Illumina (New England Biolabs, Frankfurt, Germany). The library was purified by Ampure XP Beads (Beckman Coulter, Krefeld, Germany) and measured using a Bioanalyzer (Agilent, Waldbronn, Germany). The library was paired end sequenced on a HiSeq 4000 (Illumina, San Diego, CA, USA) device, 50 million reads with a length of 100 bp.

Reads were mapped to reference genome (hg37) using STAR version 2.5.3a.

Oxford Nanopore long-read whole-genome sequencing

DNA from fresh frozen pulmonary tumor tissue was extracted by Maxwell® RSC instrument (Promega) using Maxwell® RSC Tissue DNA Kit according to the manufacturer's instructions. DNA quantities were measured using the QuantiFluor® ONE dsDNA System. Whole genome sequencing was performed with the SQK-LSK109 DNA-sequencing kit on GridION X5 instrument according to the manufacturer's protocol (Oxford Nanopore Technologies, Oxford, UK). Briefly, a library was generated from 1 μg genomic DNA without prior fragmentation and with the following modifications of the SQK-LSK109 protocol: DNA repair reaction time was set to 20 min. A library was loaded on a single flowcell R. 9.4.1. resulting in 12.6 million reads and 17.1 Gb sequencing yield. Base calling was performed with default settings on GridION X5 during the sequencing run with guppy software. The reads were aligned to the hg19 genome using ngmlr 0.2.8. long read aligner followed by processing of alignment in samtools 1.9. Coverage was calculated with BEDTools and copy number variations were profiled either in package ACE (ACE, Bioconductor, R; resolution 0.5 Mbp) or, for inspection of whole genome including sex chromosomes, genome coverage was averaged over 1 Mbp in IGVTools 2.4.17 and inspected in tdf.-format in IGV Browser. Structural variations were inspected using NanoVar software with default settings.

Targeted Exome Sequencing

DNA was extracted from an unstained formalin-fixed hepatic tumor sample and sequenced using a 50-gene NGS panel developed for clinical service at Charité – Universitätsmedizin Berlin. All genes covered by the panel are listed in

supplementary material, Table S2.

Initial *TSC1* mutation analysis

Initial *TSC1* mutation analysis was performed on DNA extracted from peripheral blood lymphocytes 10 years prior to our study by a laboratory not affiliated with our institution. Multiplex ligation-dependent probe amplification was performed; detailed information on the protocol used is not available. The results were obtained with the patient's consent.

Quantitative PCR for *TSC1* copy number analysis

Genomic DNA was extracted from fresh frozen pulmonary tumor. Quantitative PCR was performed using the HOT FIREPol EvaGreen qPCR Mix Plus (Solis Biodyne, Tartu, Estonia) and a QuantStudio No.3 device (Thermo Fisher Scientific, Hennigsdorf, Germany). The following primers were used for *TSC1* and the control loci *ALB* and *F8*: TSC1_Exon 4F TTGATTTAGACCGTGGCCCT, TSC1_Exon 4R TCTTGCAAGGTGGTCAGGAT, TSC1 Exon 11 F ACTTCCCAGGCTACTCTTTGG, TSC1 Exon 11 R TGACAGATCAGGTGGGACA, TSC1 Exon 23 F TCTGGCTGGTCTGTATCTTTCA, TSC1 Exon 23 R AAGATCTCCAATTCAAACACCTG, qhCN_AlbF TGTTGCATGAGAAAACGCCA, qhCN_AlbR GTCGCCTGTTCACCAAGGAT, qhCN_F8F CTACCATCCAGGCTGAGGTTTATG, qhCN_F8R CACCAACAGCATGAAGACTGACA.

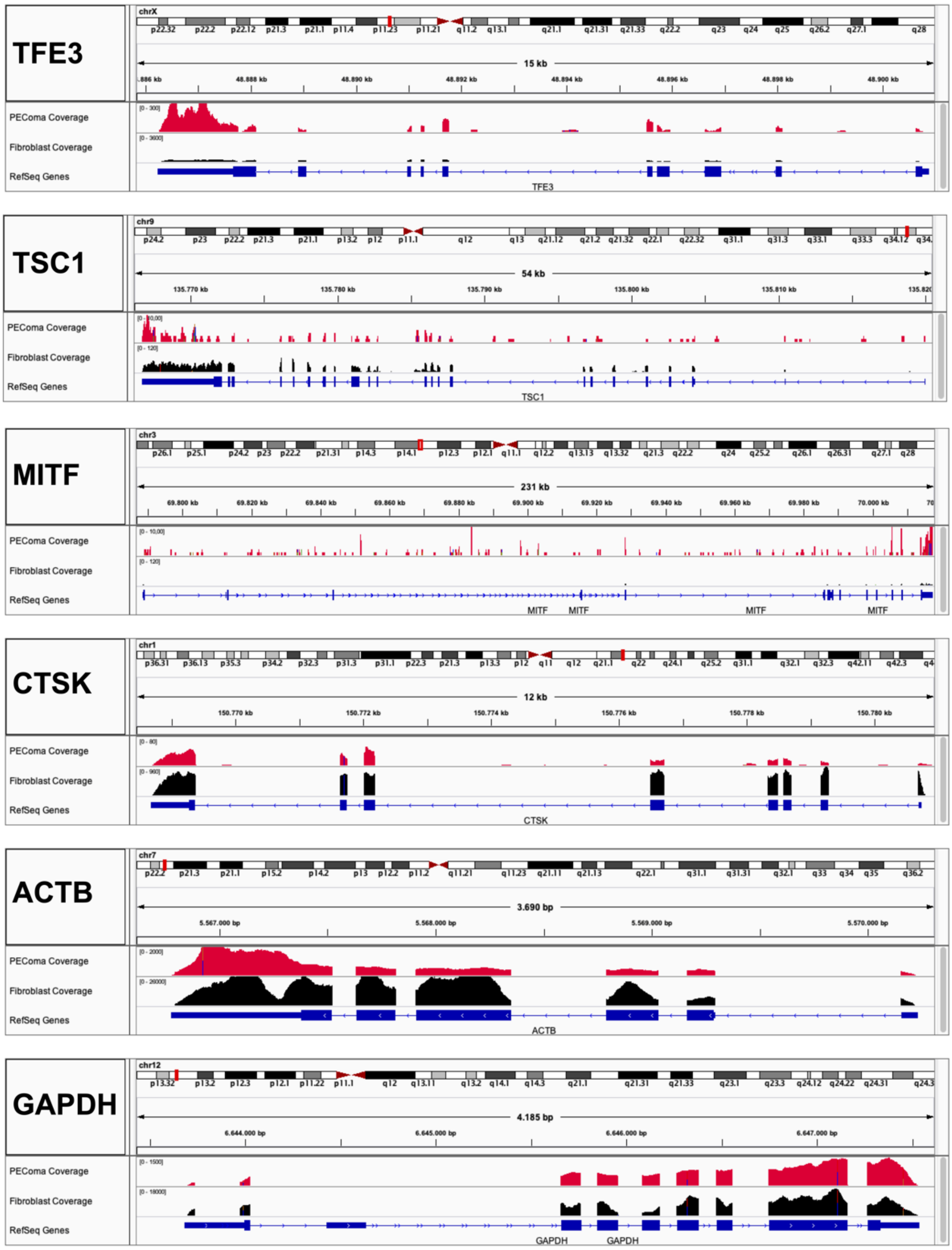

Figure S1. Expression levels of candidate genes in tumor tissue (red traces) detected by RNA-Seq compared to dermal fibroblasts (black traces). Note the pronounced reduction of coverage of 3’ exons indicating moderate mRNA quality. Sequencing traces were normalized using *ACTB* and *GAPDH* housekeeping genes as controls, which indicated 12x higher general poly A mRNA levels in dermal fibroblasts. The relevant candidate genes *TFE3*, *TSC1*, *MITF*, and *CTSK* are shown.

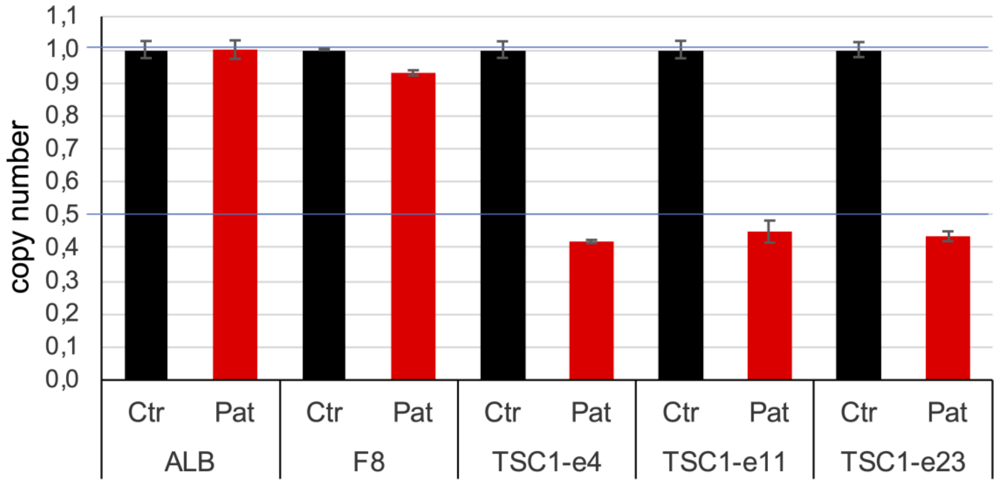

Figure S2. Results of quantitative PCR for determination of *TSC1* copy number compared to the autosomal *ALB* and the X-chromosomal *F8* gene loci. DNA from a healthy female donor was used as control (black bars). A clear heterozygous *TSC1* deletion was detected in the patient (red bars). Ctr, control; Pat, patient

Table S1. Antibodies used for immunohistochemical staining.

Antibody	Company	Clone	Dilution
HMB45	Enzo	HMB45	1:50
Melan-A	Dako	A103	1:50
Cathepsin K	Cell Marque	3F9	1:25
sm-Actin	Dako	1A4	1:400
Desmin	Dako	D33	1:50
TFE3	Cell Marque	MRQ-37	1:100

Table S2. Genes covered in the NGS panel developed for clinical service at Charité – Universitätsmedizin Berlin.

ABL1	*CTNNB1*	*FLT3*	*IDH2*	*NRAS*	*SMO*
AKT1	*EGFR*	*GNA11*	*KDR*	*PDGFRA*	*SRC*
ALK	*ERBB2*	*GNAS*	*KIT*	*PIK3CA*	*STK11*
APC	*ERBB4*	*GNAQ*	*KRAS*	*PTEN*	*TP53*
ATM	*EZH2*	*HNF1A*	*MET*	*PTPN11*	*VHL*
BRAF	*FBXW7*	*HRAS*	*MLH1*	*RB1*	*PTEN*
CDH1	*FGFR1*	*IDH1*	*MPL*	*RET*	
CDKN2A	*FGFR2*	*JAK2*	*NOTCH1*	*SMAD4*	
CSF1R	*FGFR3*	*JAK3*	*NPM1*	*SMARCB1*	
